# Safety and effectiveness of triple-drug therapy with ivermectin, diethylcarbamazine, and albendazole in reducing lymphatic filariasis prevalence and clearing circulating filarial antigens in Mombasa, Kenya

**DOI:** 10.1186/s40249-025-01282-z

**Published:** 2025-02-24

**Authors:** Christabel Khaemba, Sammy M. Njenga, Wyckliff P. Omondi, Elvis Kirui, Margaret Oluka, Anastacia Guantai, Eleni Aklillu

**Affiliations:** 1 Department of Global Public Health, Karolinska Institutet, Karolinska University Hospital, Tomtebodavägen 18A, 171 77 Stockholm, Sweden; 2 Kenya Pharmacy and Poisons Board, Nairobi, Kenya; 3 Kenya Medical Research Institute (KEMRI), Nairobi, Kenya; 4 Ministry of Health, National Neglected Tropical Diseases Program, Nairobi, Kenya; 5 Ministry of Health, National Public Health Laboratory, Nairobi, Kenya; 6 Department of Pharmacology and Pharmacognosy, School of Pharmacy, University of Nairobi, Nairobi, Kenya

**Keywords:** Active safety surveillance, Efficacy, Ivermectin, Diethylcarbamazine, Albendazole, Lymphatic filariasis, Positive participants, Adverse events, Mass drug administration

## Abstract

**Background:**

In 2018, Kenya introduced triple-drug therapy with ivermectin, diethylcarbamazine, albendazole (IDA) through mass drug administration (MDA) to accelerate the elimination of lymphatic filariasis (LF). This community-based surveillance study assessed the safety and effectiveness of IDA-MDA in reducing LF-antigenemia prevalence and circulating filarial antigens (CFA) clearance among LF infected individuals.

**Methods:**

A total of 8928 residents in Mombasa, Kenya, were screened for CFA using the Filarial Test Strip: 3464 were screened in 2018 and 5464 in 2021 after two annual IDA-MDA rounds. CFA-positive individuals in 2021 were re-tested at two and four months of post-MDA for CFA-clearance rates. Adverse events (AEs) associated with IDA-MDA were monitored via door-to-door visits on days 1, 2, and 7 post-MDA to document the incidence, type and risk factors. Efficacy outcomes included post-MDA LF-antigenemia prevalence reduction after two rounds of annual MDA and CFA clearance rate. Chi-square test compared proportions, and logistic regression analysis identified AE predictors.

**Results:**

LF antigenemia prevalence significantly decreased from 1.39% (*n* = 48) in 2018 to 0.66% (*n* = 36) in 2021 [*P* < 0.001; 95% confidence interval (*CI*) for difference in proportions: 0.003–0.012]. CFA clearance rates were 63.2% (12/19, 95% *CI:* 41.0–80.1%) at 2 months and 68.4% (13/19, 95% *CI:* 46.0–86.6%) at 4 months post-MDA. Among 53 CFA-positive individuals monitored, the cumulative 7-day AE incidence was 37.7% (95% *CI:* 25.6–51.7), higher than the general population’s 27.3% (95% *CI:* 26.4–28.2). Common AEs included nausea (11.3%), diarrhea (11.3%), abdominal pain (7.6%), and headache (5.7%). Risk factors for AEs included age, overweight status, concomitant medication use, chronic illness, and fasting before MDA.

**Conclusions:**

Triple therapy with IDA is safe and well-tolerated, with some mild-to-moderate and transient adverse events among LF-infected individuals. The high incidence of AEs highlights the need for safety monitoring during MDA. The significant reductions in LF antigenemia prevalence and high CFA clearance rates underscore IDA's effectiveness in reducing LF transmission, positioning it as a key strategy for eliminating LF as a public health problem by 2030.

**Graphical abstract:**

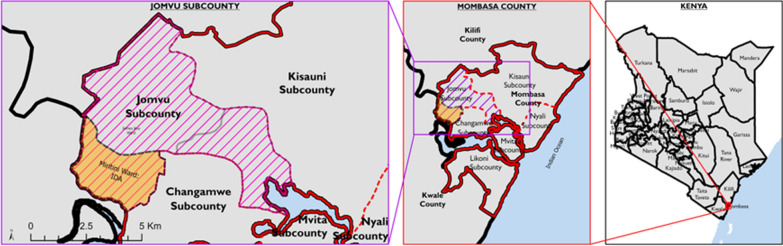

## Background

Lymphatic filariasis (LF), a mosquito-borne neglected tropia disease (NTD) caused by the filarial worms, *Wuchereria bancrofti*, *Brugia malayi* and *Brugia timori*. *W. bancrofti* is the most common etiological agent, accounting for over 90% of global infections [1]. LF ranks as the second leading cause of disability in the tropics and subtropical regions. Sub-Saharan Africa is the most affected region, with an estimated 251 million people living in areas with ongoing LF transmission [2]. Kenya is among the six most affected countries accounting for 27% of the global endemic population in 2012 [3]. In Kenya, LF is endemic in the coastal regions along the Indian Ocean, stretching from Lamu County in the north to Kwale County in the south, near the Tanzania border [4].

The World Health Organization (WHO) launched the Global Programme to Eliminate Lymphatic Filariasis (GPELF) in 2002 with the aim to interrupt transmission through periodic mass drug administration (MDA) of anti-filarial medicines and manage morbidity [5]. Despite a 74% reduction in global infections since the inception of GPELF, 51.4 million people were estimated to remain infected in 2018 [6]. By 2021, over 882 million people across 44 countries were at risk and required preventive chemotherapy [7].

The current MDA strategy involves administering repeated annual doses of albendazole (ALB) with either diethylcarbamazine (DEC) or ivermectin (IVM) for the lifespan of adult worms (about 6–8 years) [8]. Hence multiple rounds of MDA with effective coverage (≥ 65% of the total population) are required to halt transmission. The choice of drug combination largely depends on the co-endemicity onchocerciasis to avoid serious adverse events in infected individuals. In regions where onchocerciasis is co-endemic, a dual therapy of ivermectin and albendazole (IA) is employed, while diethylcarbamazine citrate and albendazole (DA) are used in areas without onchocerciasis [9].

In Kenya, LF is endemic in the six counties along the coastal region by the Indian Ocean [10]. In 2002, The Ministry of Health Kenya (MOH-Kenya) launched MDA program using DA, starting in Kilifi County. The initiative expanded to Kwale and Malindi Counties in 2003, with follow-up campaigns conducted in 2005 and 2008. In 2011, MDA was extended to Tana River and Lamu counties but was later halted due to operational challenges. Sentinel site surveys conducted in the entire region in 2015 and 2016 identified Lamu East and Jomvu sub-counties as having the highest prevalence of LF infection, with antigenemia rates of 6.3% and 6.7%, respectively [10]. These findings prompted the re-initiation of MDA across all endemic coastal counties.

After clinical trials confirmed the superior efficacy in clearing microfilariae and sterilizing adult filarial worms [11, 12], the WHO updated its guidelines in 2017, recommending a triple therapy regimen (IDA) with ivermectin, diethylcarbamazine, and albendazole combination as an alternative MDA regimen in areas where onchocerciasis is not co-endemic with LF [9]. Kenya was among the first countries to adopt and pilot triple therapy with IDA; two annual rounds were provided in 2018 and 2019 to the residents of Lamu County and Jomvu sub-county in the coast region [13].

The WHO advocates for ensuring safety and efficacy in drug surveillance as part of public health interventions. Although MDA medications are generally considered safe, several studies have reported treatment-associated adverse events (AEs) [11, 14–18]. Fear of experiencing AEs following treatment and the increased number of tablets consumed during MDA have been identified as key contributing to non-adherence. This underscores the need for rigorous safety monitoring of new drug combinations introduced in MDA programs to address concerns and build public confidence in health interventions. Continuous surveillance is essential to identify rare, severe adverse events, and detect potential drug-drug interactions.

As Kenya being the first African country to introduce triple therapy through MDA campaign, gathering real-world safety and efficacy data is crucial. During the IDA rollout, we conducted comparative safety surveillance, revealing a significantly higher incidence of mild and transient adverse events (AEs) with IDA compared to the standard DA in the general unscreened population (27.3% versus 16.2%) [15, 19]. The incidence and profile of AEs may differ in LF-infected individuals compared to the general population. Previous clinical trials indicate that individuals with higher levels of circulating filarial antigens (CFA) and microfilariae (mf) tend to experience more AEs than non-microfilaraemic individuals [16, 20, 21]. Currently, there is limited data on the safety profile of IDA among LF-infected individuals in African community settings, including Kenya. Therefore, it is crucial to monitor the safety of this intervention, particularly among vulnerable CFA-positive individuals, to facilitate timely identification and management of potential adverse effects.

Furthermore, data on the parasitological effectiveness of MDA with IDA in reducing the parasite reservoir and interrupting transmission in Africa, including Kenya, remains limited. Monitoring changes in CFA levels and microfilariae counts following MDA among infected individuals provides valuable insights into the macrofilaricidal (targeting adult worms) and microfilaricidal (targeting larval stages) effects of anti-filarial drugs [12, 22, 23]. This study aimed to assess the safety and tolerability of IDA by evaluating the incidence, type, severity, and predictors of AEs. It also examined IDA's effectiveness in reducing the prevalence of LF antigenemia in rural endemic communities after two rounds of MDA with IDA, and the CFA clearance rate among CFA-positive individuals in Kenya's coastal region.

## Methods

### Ethical statement

This study received ethical approval from the Kenyatta National Hospital-University of Nairobi Ethics and Research Committee (Ref No: KNH-ERC/A/413). Community sensitization meetings were held to inform local leaders, explain the objectives and methodology of the study, and seek community consent. Written informed consent and/or assent were obtained from adults, parents, or legal guardians of children before study enrolment.

### Study area and setting

**Fig. 1:**
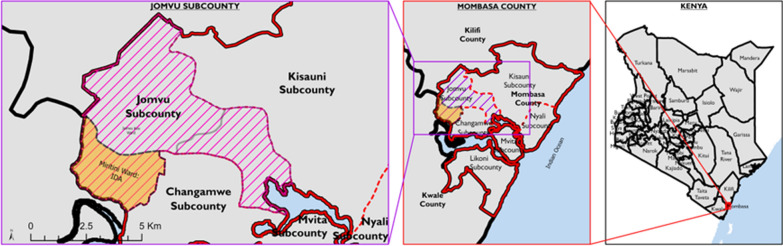
Map of the Study Area––Jomvu Sub-County (left), Mombasa County (center), and Kenya (right)

This prospective cohort study was conducted LF endemic communities between November 2018 and December 2021 in Kenya’s Coastal region in Jomvu Sub-County, Mombasa County ***(***Fig. 1). Mombasa County, located in the southeastern part of Kenya's Coastal region, covers an area of 229.9 km^2^, excluding 65 km^2^ of water mass extending 200 nautical miles into the Indian Ocean. The county comprises six sub-counties, with a projected population of 1,266,358 at the time of the study. According to the WHO, annual IDA is recommended in areas where onchocerciasis is not co-endemic with LF, where MDA has not yet started, where fewer than four effective MDA rounds have been conducted, or where MDA results have been suboptimal [9]. Because of historically high LF prevalence, irregular MDA rounds, and suboptimal coverage, Lamu County and Jomvu Subcounty were selected to pilot the triple drug therapy (IDA) in November 2018 by the MOH-Kenya. Jomvu Subcounty was chosen for our safety and efficacy surveillance study.

### Study design and population

This prospective safety and efficacy surveillance of triple therapy with IDA among LF-infected individuals was nested in a larger community-wide pharmacovigilance and impact assessment studies carried out during the IDA rollout in 2018 and a subsequent impact assessment in 2021 [13, 15, 19]. The effectiveness of IDA in reducing the prevalence of LF antigenemia within the community was assessed in 2021, following the implementation of the first and second annual MDA with IDA in 2018 and 2019, respectively.

A total of 8928 MDA-eligible residents from Jomvu Sub-County were screened for LF-antigenemia across two phases using Filarial Test Strips (FTS) [13]. The first screening conducted two weeks before IDA rollout in 2018 involved 3464 residents to establish baseline LF prevalence. In the second round, 5464 residents were screened in 2021, 17 months after the completion of two rounds of annual MDA in 2018 and 2019. With consent and approval from the NTD program, all participants who tested positive for LF antigen were identified from the NTD screening database and invited to participate in the study at a centralized location in Portreitz Sub-County Hospital.

### Treatment and follow-up

Before receiving MDA, baseline socio-demographic data including age, sex, nutritional status (assessed through anthropometric measurements), medical history, comorbidities, concomitant medications, and any pre-existing clinical symptoms (pre-MDA events) were recorded from study participants using a standard case record form. Baseline laboratory tests, including complete blood count, liver enzyme levels, urea, creatinine, and urine tests, were conducted before administering the medication.

In Kenya, all eligible residents in LF-endemic regions receive annual MDA with DA as preventive chemotherapy, following WHO and national MDA program guidelines [5, 9, 24]. In Lamu and Jomvu, the Kenyan Ministry of Health selected all MDA-eligible residents aged 2 years and older to pilot the IDA regimen. All eligible residents, including those who tested positive for CFA in our study, received a single oral dose of triple therapy: ivermectin (200 μg/kg), diethylcarbamazine (6 mg/kg), and albendazole (400 mg). This regimen was administered through directly observed therapy by community drug distributors for the general population and by healthcare workers to the CFA-positive patients during the MDA campaign, following the WHO and Kenya’s NTD program guidelines [9, 24]. The National NTD Control Program led the planning, coordination, and implementation of the MDA campaign. During the MDA campaign, residents were instructed to take their medication after eating. The study team was not involved in the planning or distribution of the drugs but adhered to the standard procedures outlined by the national MDA program.

### Effectiveness of IDA and outcome measures

Prior to MDA, all residents in the study area were screened for CFA in 2018 to establish baseline prevalence of LF-antigenemia. The same community was reassessed in 2021 after two rounds of annual IDA-MDA, with over 80% coverage, to evaluate any reduction in LF-antigenemia prevalence. Moreover, in 2021, individuals who tested positive for CFA were re-screened at two- and four-months post-MDA to assess the CFA clearance rate. CFA was detected using finger-prick blood samples applied to a Filariasis Test Strip (Alere©, Waltham, USA) as described previously [13, 25]. Two study team members independently verified positive results and re-tested for confirmation. Those testing positive for CFA were also examined for microfilariae through night-blood microscopy.

The primary efficacy outcome measures were: (i) Community-level antigenemia reduction -defined as the decrease in LF antigenemia prevalence from the pre-intervention baseline (2018) after two annual rounds of IDA-MDA, evaluated in 2021; and (ii) CFA clearance rate—defined as the proportion of CFA-positive individuals who achieved clearance (negative CFA test) at 2 and 4 months post MDA with IDA.

### Safety monitoring and outcome measures

Before receiving MDA, all participants were interviewed to document any pre-existing clinical symptoms or conditions (pre-MDA events), including fever, loss of appetite, dizziness or fainting, confusion, drowsiness, headache, cough, difficulty breathing, nausea, vomiting, diarrhea, stomach pain, itching, rash, and any other symptoms. After drug administration, active safety monitoring was conducted through door-to-door visits on days 1 and 2 to document any AEs. On day 7, participants returned to the study hospital, where any AEs were documented on-site. Participants were informed to contact data collectors to report any AEs occurring between days 3 and 6. All clinical events reported before and after MDA by the same study participant were cross-checked and verified to distinguish pre-existing clinical symptoms from MDA-associated AEs.

The primary safety outcome measure was the incidence rate of experiencing at least one type of MDA-associated adverse event (post-MDA AEs), defined as any event that occurred after drug exposure but was not reported prior to receiving the MDA. The type and severity of these adverse events served as secondary outcomes. To assess treatment tolerability, all reported AEs were categorized by severity grading using a five-level scale based on the Common Terminology Criteria for Adverse Events (CTCAE) Version 5.0 [26]. The severity grades were defined as following:

- Grade 1—Mild: asymptomatic or mild symptoms; clinical or diagnostic observations only; and intervention not indicated.
- Grade 2—Moderate: limiting age-appropriate instrumental activities of daily living (ADL). Minimal, local, or non-invasive intervention indicated.
- Grade 3—Severe: medically significant but not immediately life-threatening: disabling and limiting the self-care activities of daily living. Hospitalization or prolongation of hospitalization indicated.
- Grade 4—Life-threatening consequences: urgent intervention indicated.
- Grade 5—Death related to an AE.

### Data management and statistical analysis

Sociodemographic, clinical, and medical history, comorbidities, concomitant medications, and feeding status, were collected using a case record form and verified by the site supervisor. The data were entered into an electronic Excel sheet, verified, and re-entered for quality assurance. Cross-checking procedures were used to address any errors, incomplete entries, or missing data. Categorical variables were summarized as proportions, while continuous variables were reported as mean with standard deviation (*SD*) or median with interquartile range (IQR).

Associations between categorical variables were assessed using the Chi-square test, and predictors of adverse events were tested using univariate regression analysis. The Chi-square test was also used to compare the proportions of CFA-positive individuals before and after two rounds of IDA-MDA to evaluate the impact of the intervention. Statistical analysis was performed using STATA Version 15.1 (Stata Corp, College Station, TX, USA), with a *P*-value of < 0.05 considered statistically significant.

## Results

### Baseline characteristics of study participants

Of the total 8928 screened participants, 84 were LF-antigenemia positive, of whom 56 consented to participate in the safety surveillance study. However, only 53 participants (62.3% male and 37.7% female) were included in the safety monitoring follow-up; One participant was excluded for not being ineligible to receive MDA because of pregnancy, whereas two were excluded from the analysis due to lost to follow-up. Most of the participants were aged between 16 and 30. The median age of the participants was 34 years. The participants who reported having chronic medical conditions were 15.1%, and those on concomitant medications were 11.3%. About 17% of the participants had the presence of non-tender lymph nodes of more than 1 cm. Of the total male participants, 9.1% reported having swollen scrotums. Most participants reported normal laboratory parameters before the administration of the MDA. The sociodemographic and baseline characteristics of study participants is presented in Table 1.

**Table 1:** Socio-demographic, baseline clinical, and biochemical characteristics of study participants from Jomvu Sub-County, Mombasa, Kenya (November 2018)

Variable	Category	*N* (%)
Sex	Female	20 (37.7%)
	Male	33 (62.3%)
Age in years	2–15	13 (24.5%)
	16–30	17 (32.1%)
	31–45	11 (20.8%)
	46–60	5 (9.4%)
	61 +	7 (13.2%)
BMI	Underweight	10 (18.9%)
	Normal	15 (28.3%)
	Overweight	9 (17.0%)
	Obese	19 (35.9%)
Took concomitant medication	Yes	6 (11.3%)
	No	47 (88.7%)
Received LF medication in the previous year (2017)	Yes	22 (41.5%)
	No	31 (58.5%)
Lymphadenopathy	No lymph node > 1 cm	43 (81.1%)
	Presence of lymph nodes > 1 cm plus tender	1 (1.9%)
	Presence of lymph nodes > 1 cm plus non-tender	9 (17.0%)
Number of DEC tablets given	1–2	8 (15.9%)
	3–4	45 (84.9%)
Number of IVM tablets given	1–2	8 (15.9%)
	3–4	45(84.9%)
Chronic Illness	Yes	8 (15.1%)
	No	45 (84.9%)
Meal intake before MDA	Taken meals	35 (66.0%)
	No meals taken	18 (34.0%)
Pre -MDA events	Yes	22 (41.51%)
	No	31(58.49%)
WBC	Normal	44 (83.0%)
	Abnormal	9 (17.0%)
RBC	Normal	24 (54.7%)
	Abnormal	29 (45.3%)
Platelets	Normal	38 (71.7%)
	Abnormal	15 (28.3%)
Creatinine	Normal	37 (69.8%)
	Abnormal	16 (30.2%)
Liver function tests	Normal	26 (49.1%)
	Abnormal	27 (50.9%)
Urinalysis	Normal	31(58.5%)
	Abnormal	22 (41.5%)

*BMI* body mass index; *LF* lymphatic filariasis; *DEC* Diethylcarbamazine; *IVM* ivermectin; *MDA* mass drug administration; *WBC* white blood cells; *RBC* red blood cells

### Effect of treatment on LF antigenemia prevalence

The impact of annual MDA with IDA on reducing the prevalence of LF infection in the community was assessed after two rounds of annual MDA. A total of 8928 MDA-eligible residents from Jomvu Sub-County were screened for LF antigenemia across two rounds. In the first round, conducted two weeks prior to the 2018 IDA rollout, 3464 individuals were screened to establish baseline LF prevalence. In the second round, 5464 residents were screened during the 2021 impact assessment, 17 months after the second annual MDA. The results showed a significant reduction in LF antigenemia prevalence, with 0.66% (36 out of 3464) in 2021 compared to 1.39% (48 out of 5464) in 2018 (*P* < 0.001; 95% *CI* for the difference in proportions: 0.003–0.012).

### Effects of treatment on circulating filarial antigenemia clearance

Among the 36 individuals who tested positive for circulating filarial antigen (CFA) in 2021, 19 were available for follow-up examinations at 2 and 4 months to assess the CFA clearance rate after receiving MDA. Of the 19 CFA-positive individuals, 12 tested negative for CFA at two months of post MDA, and 13 tested negative at four months post-MDA. Those who tested CFA-negative at 2 months of post-MDA remained negative at 4 moth post MDA. This resulted in CFA clearance rates of 63.2% (12 out of 19, 95% *CI:* 41.0–80.1%) at two months and 68.4% (13 out of 19, 95% *CI:* 46.0–86.6%) at four months of post-MDA.

### Treatment associated adverse events

**Fig. 2:**
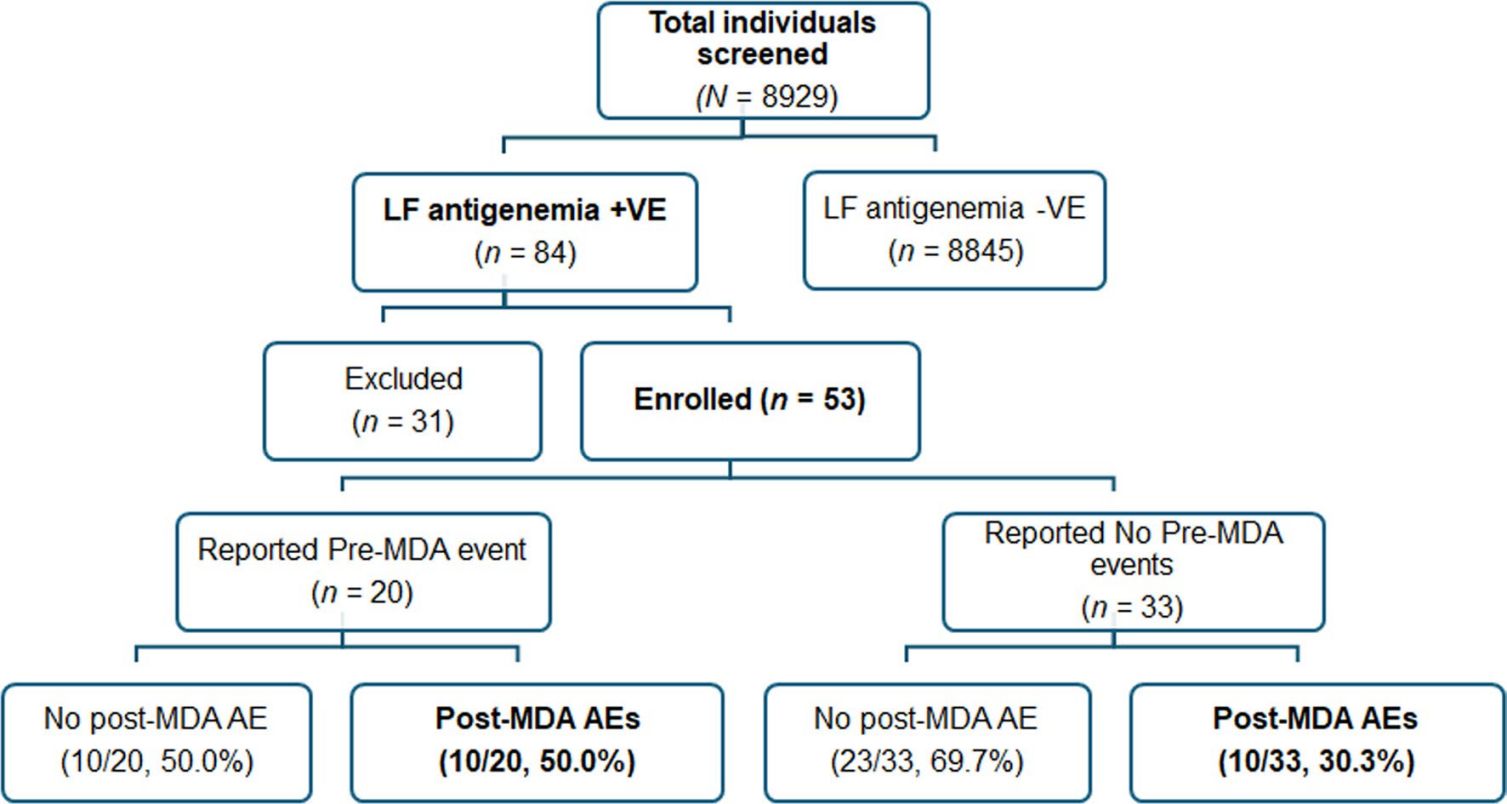
Study flowchart depicting the pre-screening and enrollment process of LF antigenemia positive (LF antigenemia + ve) individuals, along with the outcome of adverse event monitoring following mass drug administration with IDA (Post-MDA AEs)

Among the 53 LF-infected (CFA-positive) participants monitored for safety, 20 reported experiencing at least one type of adverse event post-MDA with IDA. The cumulative incidence of experiencing at least one type of post-MDA AEs over the seven-day follow-up period was 37.7% (95% *CI:* 25.6–51.7%). Participants who reported pre-MDA clinical symptoms (events) had a higher incidence of post-MDA AEs compared to those who did not report events before receiving MDA (50.0% vs. 30.3%) (Fig. 2). The overall incidence rate of AEs among LF-infected was higher than that observed in the general unscreened population who received IDA as preventive chemotherapy to control LF in the same study area (37.7% versus 27.3%, 95% *CI:* 26.4–28.2%). Details of the incidence and risk factors of IDA-associated AEs in the general unscreened population have been presented previously [19]. Table 2 presents the severity grading of reported AEs following MDA among individuals infected with lymphatic filariasis. The majority of post-MDA AEs were mild (96.2%), while a small proportion were moderate (3.9%). Notably, no severe adverse events were reported..

**Table 2:** Severity grading of adverse events following mass ivermectin, diethylcarbamazine and albendazole administration in Lymphatic filariasis infected participants from Jomvu Sub-County, Mombasa, Kenya (November 2018)

Adverse events	Number of events	Severity grading		
		Grade 1 (Mild)	Grade 2 (Moderate)	Grade 3 (Severe)
Nausea	5	5 (100.0%)		
Stomach Pain	3	3 (100.0%)		
Diarrhoea	6	5(83.3%)	1(16.7%)	
Headache	3	3 (100.0%)		
Dizziness	2	2 (100.0%)		
Loss of appetite	2	2 (100.0%)		
Fever	1	1(100.0%)		
Drowsiness	2	2 (100.0%)		
Confusion	1	1(100.0%)		
Cough	1	1(100.0%)		
Total	26	25 (96.2%)	1 (3.8%)	0 (0.0%)

**Fig. 3:**
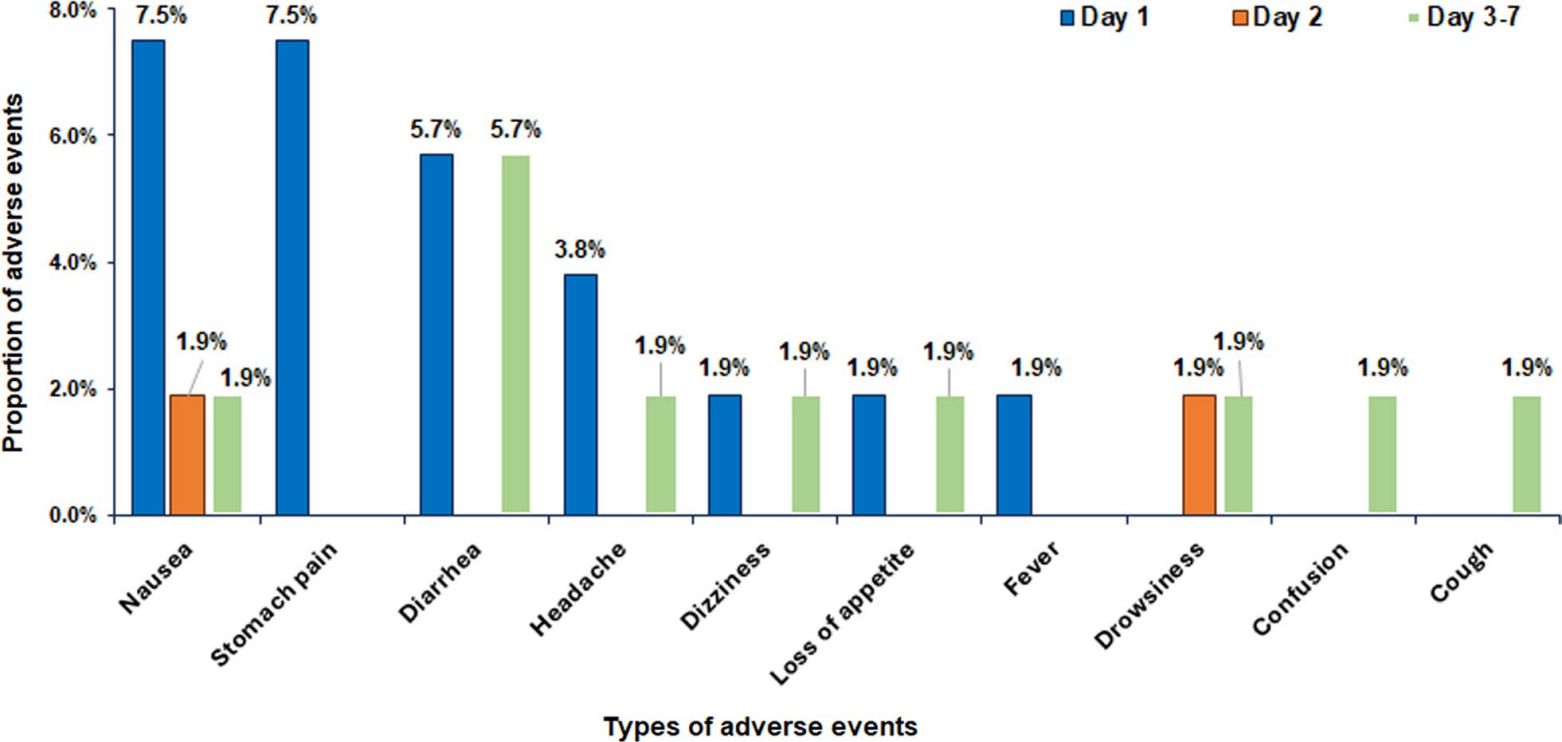
Proportion of MDA-associated adverse events (AEs) following triple therapy (IDA) stratified by type of the days of occurrence

Figure 2 presents the study flow chart detailing the recruitment process, follow-up and incidence of AEs among LF-infected patients**.** A total of 28 AEs were reported by 20 study participants. The proportion of those who reported one type of AE was 26.4% (*n* = 14), and those who reported two or three events were 3.8% (*n* = 4) and 7.5% (*n* = 2) respectively. Most reported AEs occurred on day 1 and resolved within a week (Fig. 3).

**Fig. 4:**
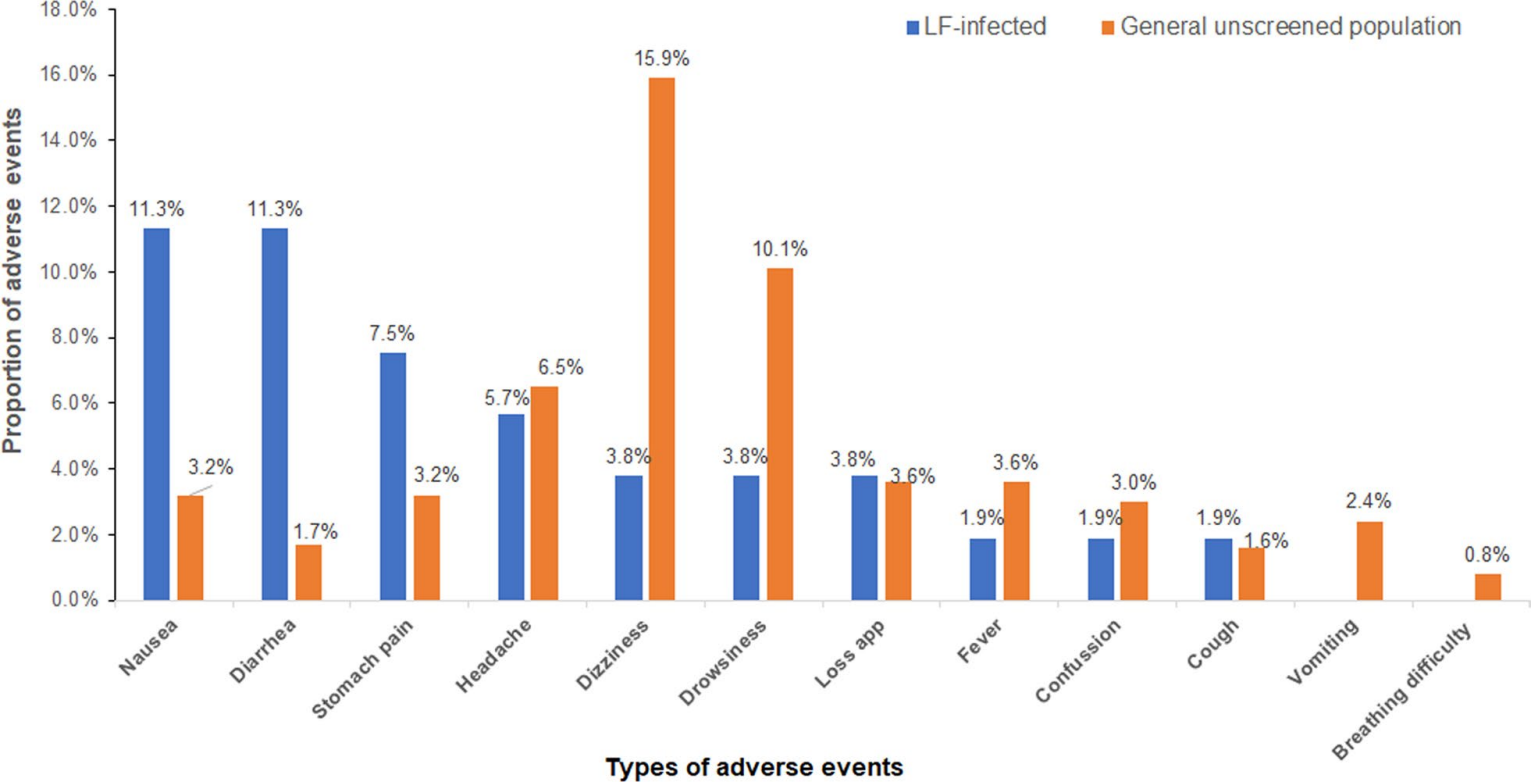
Comparison of the incidence and type of AEs associated with post-MDA using IDA between LF-infected individuals versus the general unscreened population

The four most common type of post-MDA AEs were nausea (11.3%, *n* = 6), diarrhea (11.3%, *n* = 6), stomach pain (7.6%, *n* = 4), and headache (5.7%, *n* = 3) in contrast dizziness (15.9%, *n* = 1657), drowsiness (10.1%,* n* = 1053), and headache (6.5%, *n* = 678) were the three most reported AEs among the general unscreened population. Interestingly, no vomiting or difficulty in breathing was reported amongst the LF-positive participants. The comparison of the proportions of AEs between LF-positive study participants and the general unscreened population is illustrated in Fig. 4.

A total of 26 out of the 28 reported AEs (92.8%) were graded for severity. No participant reported an AE with a severity scale more than Grade 2. Twenty-five (96.2%) of the AEs were graded as mild, while 3.9% (*n* = 1) were graded as moderate. There were no severe or serious AEs.

### Factors associated with the occurrence of adverse events

The likelihood of experiencing AEs was slightly higher among male participants compared to female participants (39.4% vs. 35.0%). Participants aged 16 to 30 were more likely to report AEs (40.0%) than those in other age categories. Additionally, an increase in the number of DEC or IVM tablets taken was associated with a higher occurrence of AEs.

Other factors linked to the occurrence of AEs included being overweight or obese, taking concomitant medications, having a chronic illness, not having eaten a meal before receiving MDA, and having pre-existing clinical symptoms (pre-MDA events). Participants with abnormal baseline values in their red blood cells (RBCs) and creatinine levels were also more likely to experience AEs. However, none of these variables were found to be statistically significant (Table 3).

**Table 3:** Factors associated with adverse events following mass drug administration with IDA among LF-infected individuals from Jomvu Sub-County, Mombasa, Kenya (November 2018) using chi-square test

Variable	Adverse event		
	**No (*n* = 33)**	**Yes (*n* = 20)**	***P*-value**
	***n* (%)**	***n* (%)**	
Sex			
Female	13 (65.0)	7 (35.0)	0.75
Male	20 (60.6)	13 (39.4)	
Age in years			
2–15	9 (27.3)	4 (20.0)	0.12
16–30	9 (27.3)	8 (40.0)	
31–45	8(24.2)	3 (15.0)	
46––60	2 (6.1)	3 (15.0)	
61 +	5 (15.2)	2 (10.0)	
BMI			
Underweight	8 (80.0)	2 (20.0)	0.20
Normal	10 (66.7)	5 (33.3)	
Overweight	3 (33.3)	6 (66.7)	
Obese	12 (63.2)	7 (36.8)	
Took concomitant medication			
Yes	3 (50.0)	3 (50.0)	0.48
No	30 (63.8)	17 (36.2)	
Received MDA in the previous year (2017)			
Yes	13 (59.1)	9 (40.9)	0.69
No	20 (64.5)	11(35.5)	
Number of DEC tablets given			
1–2	6 (75.0)	2 (25.0)	0.42
3–4	27 (60.0)	18 (40.0)	
Number of IVM tablets given			
1–2	6 (75.0)	2(25.0)	0.42
3–4	27 (60.0)	18 (40.0)	
Chronic illness			
Yes	8 (66.7)	4 (33.3)	0.72
No	25 (60.9)	16 (39.2)	
Meal intake before MDA			
Taken meals	23 (65.7)	12 (34.3)	0.47
No meals taken	10 (55.6)	8 (44.4)	
Pre–MDA events			
Yes	12 (54.5)	10 (45.5)	0.33
No	21 (67.7)	10 (32.3)	
WBCs			
Normal	27 (61.7	17 (38.6)	0.77
Abnormal	6 (66.7)	3 (33.3)	
RBCs			
Normal	18 (75.0)	6 (25.0)	00.8
Abnormal	15 (51.7)	14 (48.3)	
Platelets			
Normal	23(60.5)	15 (39.5)	0.68
Abnormal	10 (66.7)	5 (33.3)	
Creatinine			
Normal	18 (69.2)	8 (30.8)	0.31
Abnormal	15 (55.6)	12 (44.4)	
LFTs			
Normal	18 (69.2)	8 (30.8)	0.31
Abnormal	15 (55.6)	12 (44.4)	
Urinalysis			
Normal	20 (64.5)	11 (35.5)	0.69
Abnormal	13 (59.1)	9 (40.9)	

*DEC* diethylcarbamazine, *WBCs* White blood cells, *RBC* Red blood cells, *LFTs* Liver Function Tests, *IDA* ivermectin, diethylcarbamazine, albendazole combination, *MDA* mass drug administration

### Predictors of adverse events

The risk factors associated with adverse events following the administration of IDA amongst the LF-positive participants were analyzed using a univariate logistic regression model. Age, concomitant medication, number of DEC and IVM tablets, having pre-MDA, events, impaired WBC, and platelets were possible predictors of the occurrence of events. However, none of them was statistically significant and, therefore, not possible to subject the variables to a multivariate generalized linear binomial regression (Table 4).

**Table 4:** Predictors of adverse events following mass drug administration with IDA among LF-infected individuals using univariate logistic regression analysis

Variable	Crude risk ratios	*P*‐value	95% *CI*
Sex			
Female	1	0.75	0.541–2.340
Male	1.13		
Age in years			
2–15	0.64	0.28	0.253–1.499
16–20	0.41	0.32	0.067–2.394
21–64	1		
65–99	1.00		
BMI			
Normal	1		
Underweight	0.6	0.48	0.143–2.510
Overweight	2	0.11	0.853–4.688
Obese	1.11	0.83	0.437–2.792
Concomitant medication			
No	1	0.47	0.570–3.352
Yes	1.38		
Participated in MDA in the previous year (2017)			
Yes	1	0.69	0.578–2.301
No	1.15		
Number of DEC tablets given			
1–2	1		
3–4	1.6	0.46	0.457–5.598
Number of ivermectin tablets given			
1–2	1		
3–4	1.6	0.46	0.457–5.598
Chronic illness			
No	1	0.73	0.352–2.074
Yes	0.85		
Meal intake before MDA			
No meal	1	0.462	0.427–3.657
Taken meal	0.77		
Reported pre-MDA events			
No	1	0.33	0.710–2.796
Yes	1.4		
WBC			
Normal	1	0.77	0.428–3.137
Abnormal	1.16		
RBC			
Normal	1	0.10	0.235–1.139
Abnormal	0.52		
Platelets			
Normal	1	0.68	0.523–2.680
Abnormal	1.18		
Creatinine			
Normal	1	0.98	0.474–2.148
Abnormal	1.01		
LFTs			
Normal	1	0.31	0.339–1.414
Abnormal	0.6923		
Urinalysis			
Normal	1	0.69	0.435–1.731
Abnormal	0.8673		

*BMI body mass index, DEC* diethylcarbamazine, *WBCs* White blood cells, *RBC* Red blood cells, *LFTs* Liver Function Tests, *IDA* ivermectin, diethylcarbamazine, albendazole combination, *MDA* mass drug administration

## Discussion

This community-based safety and efficacy surveillance of preventive therapy with IDA was conducted in Kenya’s LF-endemic Jomvu sub-county. Our key findings include: (i) Substantial reduction in LF antigenemia prevalence: Two annual rounds of MDA with IDA achieved a 63% reduction in community antigenemia prevalence, with CFA positivity declining from 1.39% in 2018 to 0.66% in 2021; (ii) Rapid antigen clearance: among CFA-positive individuals, clearance rates reached 63.2% at two months and 68.4% at four months post-MDA, demonstrating robust treatment efficacy; and (iii) Safety profile: CFA-positive participants experienced a higher incidence of mild-to-moderate and transient AEs (37.7%) compared to the general population (27.3%) receiving the same therapy [19], though no severe adverse events were reported. The regimen was well-tolerated overall, reinforcing its feasibility for large-scale implementation [19]. To our knowledge, this is the first community-based surveillance report in sub-Saharan Africa to assess the safety and effectiveness of IDA therapy in reducing CFA positivity following the implementation of a triple-drug therapy in MDA program.

The circulating filarial antigen serves as a sensitive biomarker for detecting viable *W. bancrofti* adult worms [27, 28], and the macrofilaricidal activity of anti-filarial drugs can be indirectly assessed through post-treatment changes in CFA levels [22]. Our result indicates that the implementation of two rounds of IDA as preventive chemotherapy markedly reduced the prevalence of LF antigenemia in the community. The CFA positivity rate dropped significantly, from 1.39% in 2018 to 0.66% in 2021. This decline reflects the effectiveness of the MDA strategy in targeting and reducing the transmission of LF within the population. These results underscore the value of an ongoing MDA program with IDA in achieving long-term control and potential elimination of LF, especially when combined with regular monitoring and follow-up assessments to track the impact of interventions.

The high CFA clearance rate among individuals who initially tested positive for antigenemia further highlights the success of the intervention. At two months post-MDA, 63.2% of antigenemia-positive individuals showed no detectable CFA, indicating a robust early response to treatment. By four months post-MDA, this clearance rate increased to 68.4%, demonstrating sustained progress in reducing the parasite load in those affected. The improvement over time suggests that the IDA regimen is effective in reducing transmission at the community level and clearing existing infections in individuals. A recent study from Tanzania reported a high clearance rate of microfilaremia but a low clearance rate of filarial antigenemia (12.6%) at 6 months following dual therapy with ivermectin and albendazole (IA) [29]. The observed high CFA-clearance rates of IDA (63.2% at two- and 68.4% at four-month post-MDA) in our study indicates superior macrofilaricidal effect of IDA compared to IA. Our results are consistent with previous reports showing that IDA is more effective than IA in inactivating adult worms, and both treatments were well tolerated, with no serious adverse events reported [23].

Previous studies have shown that IDA is more effective at clearing microfilariae after the first round of MDA, though reductions in CFA levels occur more gradually over the first two years [20, 30]. While our post-treatment CFA retesting time point differ from those in other studies, our results support the idea that anti-filarial drugs typically have limited macrofilaricidal effects on adult worms [31]. However, the significant reduction in CFA positivity observed in our study indicates that IDA not only sterilizes adult worms but also exhibits enhanced macrofilaricidal activity. Thus, administering two or more rounds of MDA with IDA could significantly reduce LF transmission in Kenya.

The incidence of experiencing one, two, and three or more types of AEs was 26.4%, 3.8%, and 7.5%, respectively. The study participants tolerated IDA triple therapy well. This was consistent with other published studies, where most of the AEs reported by the LF-positive participants receiving IDA were mild to moderate and transient [15, 19, 32]. Sex differences, pre-existing clinical symptoms, having out-of-range values in WBCs, RBCs, and platelets, and chronic illness were risk factors associated with AEs post-MDA with IDA preventative chemotherapy; however, none of these factors was statistically significant.

Previous studies on various drug combinations used for preventive chemotherapy in LF elimination and control have reported common AEs such as dizziness, drowsiness, fever, and confusion, among others [11, 15, 19, 23, 33]. In our study, most reported AEs occurred within the 48 h post-MDA and were mild and transient, resolving by day seven. A notable difference in AE profiles emerged between study cohorts: Systemic AEs such as dizziness, drowsiness, and headache were more prevalent in the general un-screened population, whereas gastrointestinal AEs, such as nausea, diarrhea and stomach pain were more frequently reported among LF-infected (Fig. 4). This variation may be due to underlying health conditions in LF-infected individuals. Chronic systemic inflammation and immune dysregulation associated with LF infection could increase susceptibility to gastrointestinal disturbances, potentially explaining the higher incidence of nausea and vomiting in this cohort. Additionally, direct gastrointestinal irritation caused by ALB and IVM—both known to induce nausea, vomiting, and diarrhea—may further contribute to these findings [34]. Understanding these differences is crucial for optimizing AE management strategies and providing targeted clinical support to patients during treatment.

The incidence of MDA-associated AEs from the literature varies widely, ranging from 12% to about 61.1%, with some studies indicating that individuals with microfilaremia participants are likely to experience more AEs [19, 23, 32]. Research also suggest that CFA-positive participants treated with IDA experience a higher frequency of AEs than those treated with either DA or IA. This has been attributed to immunological reactions triggered by dying parasites, as IDA demonstrates greater efficacy than dual therapies [20, 23]. In our previous study on un-screened general populations, we reported a higher incidence rate of AEs among the IDA group (27.3%) compared to the dual DA group (16.2%). In current study, the incidence rate of AEs following IDA-MDA among LF-infected participants was 37.7%, exceeding that of the unscreened population and aligning with findings from other studies with the other studies [15, 19]. Prior research on the safety of antifilarial drugs has documented that most reported AEs are mild and moderate, requiring little or no intervention. This study has replicated this, with 96.2% of all graded AEs categorized as mild. The results are consistent with the study conducted in the general populations in Kenya using either dual DA or IDA triple therapies [15, 19].

Although not statistically significant, sex differences were associated with AEs, with males reporting higher incidences of AEs than female participants. However, our findings in the present paper differ from previous studies that reported a higher frequency of AEs in female participants, including our community-based survey on IDA [14, 16, 19, 33, 35]. The discrepancies between our results and those of prior studies could be attributed to two key factors: the gender imbalance in our participant cohort (with a higher proportion of males) and the limited sample size. The disparity in treatment-related AEs rates between males and females may be due to pharmacokinetic variability, which influences drug metabolism and physiological responses. Studies suggest that females experience AEs at twice the rate of males, reinforcing the role of pharmacokinetic differences in these variations [36–38]. Furthermore, other factors, such as social and health-seeking behavior, can lead to gender-related differences in the reporting of AEs [37]. There was no significant difference in the occurrence of AEs across different age categories. However, an increased frequency of AEs was associated with higher doses of IVM and DEC tablets. This finding aligns with previous studies that have documented an additive interaction between IVM and DEC, leading to immunological reactions following the death of filarial parasites in the body [39].

Baseline hematological and biochemical parameters were assessed before MDA. The results suggested that impaired baseline WBC and platelets were associated with the occurrence of AEs, though this association did not reach statistical significance. While reports on the effects of IDA on hematological parameters are scarce, one study comparing the efficacy of IVM and DEC against naturally infected *Brugia malayi* microfilaria in dogs reported mild anemia, leukocytosis, and thrombopenia in microfilaraemic animals. A similar study on the effect of IVM on hematological and biochemical parameters in goats reported a remarkable reduction in the WBCs, RBCs and an increase in the liver enzymes, alkaline phosphatase, bilirubin, and kidney functions [40]. Therefore, participants with abnormal levels of hematological parameters were more likely to report AEs. More studies need to be conducted on the human population to elucidate the effect of IDA on hematological and biochemical parameters.

While this study provides critical insights, certain limitations merit consideration. The relatively small number of CFA-positive individuals (*n* = 53), despite community-wide screening of > 8000 individuals, reflects the epidemiological success of sustained MDA in reducing LF burden, posing inherent challenges for recruiting LF-positive cases in settings nearing elimination. Additionally, since we recruited positive participants from an NTD program survey, we were unable to compare the safety of IDA versus DA in CFA-positive group. A key strength of our study is its longitudinal repeated measures design, which allowed individuals or communities to serve as their own controls when assessing treatment impact. We also implemented active cohort event monitoring to closely track participants for seven days after treatment. As the first baseline study in the coastal region of Kenya and sub-Saharan Africa to evaluate the safety of IDA in LF-positive individuals, our findings lay the groundwork for future large-scale studies.

## Conclusions

Triple therapy with IDA was generally well-tolerated among LF infected individuals, with most AEs being mild to moderate and transient. However, the higher incidence of AEs among CFA-positive participants underscores the need for safety monitoring during MDA campaigns. Our study confirms that annual MDA with IDA effectively reduces LF antigenemia prevalence after two rounds in a community setting. The significant post-MDA clearance rates of CFA demonstrate a strong macrofilaricidal effect. These results validate IDA’s capacity not only to sterilize adult *W. bancrofti worms* but also to disrupt transmission dynamics, surpassing the efficacy of conventional dual-therapy regimens. Given its superior efficacy compared to dual therapy, repeated rounds of IDA-based MDA could significantly accelerate LF elimination in Kenya and other endemic regions. The observed reduction in LF antigenemia prevalence in the community and the high CFA clearance rates among infected collectively underscore IDA’s potential as a key public health intervention for achieving LF elimination by 2030.

## Data Availability

All data generated or analyzed during this study are included in the manuscript.

## Abbreviations

AEs: Adverse events
BMI: The body mass index
*CI*: Confidence interval
GPELF: Global Programme to Eliminate Lymphatic Filariasis
IA: Ivermectin with albendazole combination
DA: Diethylcarbamazine and albendazole combination
IDA: Tiple therapy with ivermectin, diethylcarbamazine and albendazole combination
LF: Lymphatic filariasis
MDA: Mass drug administration
NTD: Neglected tropical diseases
SSA: Sub-Saharan Africa
WHO: World Health Organization
